# Reconstruction of deep and perforating corneal defects in dogs—A review (Part II/III): Biomaterials and keratoprosthesis

**DOI:** 10.1111/vop.13287

**Published:** 2024-10-02

**Authors:** Eric C. Ledbetter, Rick F. Sanchez, Marta Leiva Repiso

**Affiliations:** ^1^ DVM, DACVO, Department of Clinical Sciences, College of Veterinary Medicine Cornell University Ithaca New York USA; ^2^ EBVS® Specialist, Dipl ECVO, DVM, CertVetEd/FHEA Specialistische Dierenkliniek Utrecth (SDU‐AniCura) Utrecht The Netherlands; ^3^ EBVS®Specialist, Dipl ECVO, PhD, DVM, Servei d’Oftalmologia, Hospital Clínic Veterinari, Campus Universitat Autònoma de Barcelona, Spain; Departament de Medicina i Cirurgia Animal, Facultat de Veterinària Universitat Autònoma de Barcelona Barcelona Spain

**Keywords:** bioengineered cornea, corneal stroma xenograft, corneal thickness, recommendation, small intestinal submucosa graft, urinary bladder acellular matrix

## Abstract

The surgical reconstruction of severe corneal ulcers is a common and crucial component of the clinical practice of veterinary ophthalmology. Numerous surgical techniques are used in dogs for corneal reconstruction, and these techniques may be categorized by the material used to repair the corneal lesion. The first part of the present review described procedures that utilize autogenous ocular tissues, homologous donor tissues, and heterologous donor tissues. In this second part of the review, the categories of biomaterials and keratoprosthetics will be summarized. Biomaterials that are reported for use in dogs include amniotic membrane, porcine urinary bladder acellular matrix, porcine small intestinal submucosa, acellular porcine corneal stroma, and other miscellaneous soft tissue and cartilage grafts (e.g., preserved equine renal capsule, autologous omentum, autologous buccal mucosa membrane, bovine pericardium, and homologous peritoneum). Descriptions of keratoprosthesis surgery in dogs are currently limited, but the use of artificial corneal transplants hold promise for dogs with severe, vision‐compromising corneal disease that is not amenable to other reconstruction techniques. This review describes the results of experimental studies evaluating these graft materials in dogs, and it will summarize the findings and outcomes of the clinical articles published in each material category. Reporting inconsistencies and areas where additional research is required will be highlighted to help guide future studies in this area. A major aim of this review is to help identify potential subjects that could be evaluated in future investigations and that might lead to refinements in clinical practice.

## 1 INTRODUCTION

This second part of the review of corneal reconstructive techniques in dogs focuses on the use of biomaterials and keratoprosthesis for corneal reconstruction in this species. A literature review was performed to identify source material using the same search strategy defined in the first part of this review on corneal reconstructive techniques in dogs. The aims of the review include to report the findings and outcomes of the articles published in each category, to identify the potential challenges that specialists find when writing about corneal reconstruction so that these may be minimized in future studies, and to identify knowledge gaps and encourage prospective studies on these areas.

A search of the peer reviewed veterinary literature written in English in the last 61 years (i.e., start of 1962 to the end of 2023) and that focused on corneal reconstruction for spontaneous corneal ulcerative disease of various causes in canine patients was carried out to find out how many articles existed. The author employed a commonly used a search engine (i.e., PubMed) and included terms such as ‘corneal ulcerative disease’, ‘corneal reconstruction’, ‘corneal surgery’, ‘corneal graft’, ‘conjunctival pedicle flap’ (and graft), ‘conjunctival patch graft’, ‘corneolimboconjunctival transposition’, ‘corneoconjunctival transposition’, ‘third eyelid graft’, ‘buccal mucosal graft’, ‘biomaterial’, ‘amnion’, ‘renal capsule’, ‘bovine pericardium’, ‘porcine intestinal submucosa’, ‘Biosist’, ‘porcine urinary bladder’, ‘keratoplasty’ (lamellar and penetrating), ‘corneal melting’, and/or ‘keratoprosthesis’, combined with the terms ‘dog’ or ‘canine’. Cross referencing was also carried out.

## 2 BIOMATERIALS

**FIGURE 1:**
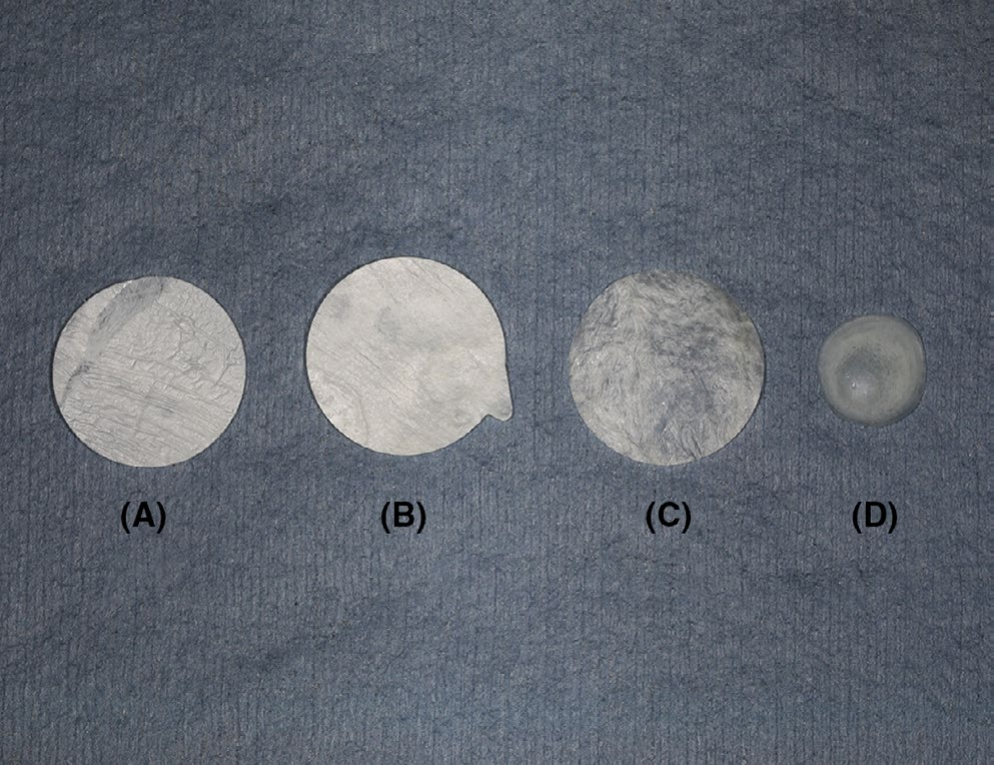
Examples of dehydrated biomaterial products used for corneal reconstruction in veterinary ophthalmology: (A) 15 mm amniotic membrane disc (Vetrix® EyeQ), (B) 15 mm porcine urinary bladder acellular matrix disc (ACell® Vet), (C) 15 mm porcine small intestinal submucosa disc (Vetrix® BioSIS), (D) 10 mm acellular porcine corneal stroma disc (BioCorneaVet™).

Numerous bioengineered materials have been employed in veterinary ophthalmology as graft materials for corneal reconstruction (Figure 1). These materials serve as scaffolds for cellular migration, provide tectonic tissue support, and are variably transparent, biocompatible, and biodegradable.^1^ For the purposes of this review, biomaterials have been divided into the following categories: amniotic membrane, porcine urinary bladder acellular matrix, porcine small intestinal submucosa, acellular porcine corneal stroma, and miscellaneous soft tissue and cartilage grafts (e.g., preserved equine renal capsule, autologous omentum, autologous buccal mucosa membrane, bovine pericardium, and homologous peritoneum). These are covered in this section, while grafts with autologous ocular materials are covered in a section dedicated to those tissues.

### 2.1 Amniotic membrane

Amniotic membrane (AM) is the inner layer of the fetal membranes and consists of three distinct layers: a monolayer of epithelium, the basement membrane, and the stroma. Amniotic membrane contains cytokines, proteoglycans, collagen, laminin, and fibronectin and serves as a substrate for the growth, migration, and adhesion of epithelial cells.^2^ Amniotic membrane in inherently transparent lacks immunogenicity and possess numerous characteristics which can be exploited during ocular surface reconstruction including anti‐inflammatory, antifibrotic, antiprotease, antiangiogenic, and antimicrobial properties.^2^, ^3^

Amniotic membrane grafting materials have been prepared from numerous species including human, equine, canine, bovine, rabbit, and porcine sources. Cryopreservation in storage media (such as glycerol) and lyophilization are frequently used for preservation. Cryopreservation often requires the use of specialized equipment, such as ultra‐low temperature freezers, for preservation and maintenance of these conditions can be logistically complicated for shipment and storage. Commercially available products include human (Ambio® and Omnigen®) and bovine (AmnioVet® and Vetrix® EyeQ) origin graft materials that are supplied in multiple different forms including sheets and discs. Some amnion grafts include the chorion layer for added thickness and structural support. In general, AM grafts can be applied either epithelial side up (i.e., inlay technique) to serve as a scaffold for migrating epithelial cells over the membrane and to facilitate graft integration into the corneal tissues, or stromal side up (i.e., overlay technique) as a biological patch to cover lesions without integration of the graft, protect the corneal surface, and to act as a barrier to prevent tear film leukocytes and other components from accessing the cornea.^3^ The “inlay technique” may be used with a single layer of AM that is typically sutured to the edge of the ulcer, or in an “inlay‐stacked technique” of multiple layers, where only the last layer is typically sutured onto the edge of the ulcer.^4^, ^5^, ^6^, ^7^, ^8^, ^9^, ^10^, ^11^ The “inlay” and “overlay” techniques may be also used together in a combined technique, and surgeons may choose to suture the inlay onto the wound's edge or not. The AM in the “overlay” technique is usually sutured onto the limbus or perilimbal conjunctiva.^4^, ^5^, ^6^, ^7^, ^8^, ^9^, ^10^, ^11^

Several studies have evaluated the use of AM transplantation in dogs with experimentally created lamellar and full‐thickness corneal wounds and investigated a range of parameters from the clinical healing response to histologic changes found over time. Glycerin‐preserved equine AM grafting was evaluated following experimental superficial anterior lamellar keratectomy in dogs.^4^ A 5.0 mm trephine was used to create a superficial keratectomy, and an AM graft was suture to the defect. Graft orientation (i.e., inlay or overlay technique) was not described. Clinical and histopathologic evaluation of the eyes was performed between postoperative Days 2 and 60. Acutely, corneal edema was present adjacent to the graft, and progressive corneal vascularization was observed and then slowly regressed. At 60 days, postoperative, corneal fibrosis was clinically evident at the surgical site. Histologically, the grafts were well integrated into the corneal tissue.^4^

Glycerin‐preserved porcine AM transplantation was evaluated in dogs for the repair of experimental, 0.4 mm deep corneal defects created in the axial cornea using an 8‐mm radial vacuum trephine.^5^ The AM was placed epithelial side up into the corneal defect and sutured to the adjacent cornea. Another piece of AM was then placed over the entire cornea and sutured to the conjunctiva. Dogs were clinically evaluated, and samples collected for transmission electron microscopy evaluation of corneal microstructure and analysis of the relative ratio of glycosaminoglycans, at intervals for up to 40 days after surgery.^5^ Severe corneal opacification and vascularization were noted in the first 20 postoperative days, but near complete corneal clarity was restored by postoperative Day 40. On postoperative Day 40, only a small amount of AM remained in the corneal stroma and corneal thickness, parenchymal cell number, mean collagen fibril diameter, collagen fibril content, and glycosaminoglycans ratio had largely normalized.^5^

The effects of bovine freeze‐dried AM transplantation were evaluated in eight normal Shih‐tzu dogs with experimentally‐created superficial corneal erosions.^6^ An 8.0 mm trephine was used to a make a 62.5 μm deep corneal incision, and 100% ethanol was applied to the area to remove the epithelium. Amniotic membrane grafts were sutured to the bulbar conjunctiva (graft orientation was not described) and compared to dogs treated with nictitating membrane flaps, contact lens placement, or no further treatment. Corneal healing and a proliferation cell nuclear antigen assay were evaluated after 48 h. Healing rates and epithelial cell proliferation were found to be highest in the AM group compared to the other treatment regimes.^6^

An experimental study evaluated the use of glycerol‐preserved equine AM for the repair of full‐thickness canine corneal defects.^7^ Perilimbal, full‐thickness, 5.0 mm square corneal defects were created using a scalpel blade, and similar‐sized grafts of AM were sutured into the defect (graft orientation was not described). Clinical and histopathologic evaluation of the eyes was performed between postoperative Days 2 and 180. No aqueous humor leakage was detected around the bulging grafts acutely. Extensive fibrovascular tissue begins to infiltrate the graft region after 7 days and then was noted to be regressing by postoperative Day 30.^7^ At study conclusion on postoperative Day 180, dense corneal fibrosis and vascularization were present in the graft area. During histopathologic evaluation of the corneas, there was an acute inflammatory phase followed by vascularization and fibroblast proliferation development around the graft site that persisted for several weeks. By postoperative Day 180, normal corneal architecture was histologically restored at the site of the implant and remnants of the implanted AM remained visible.^7^

A total of 196 clinical canine eyes from patients with a variety of ophthalmic diseases, including multiple corneal ulcers and perforations, as well as reconstruction after seven dermoid removals, two epithelial inclusion cysts, and a single case each of ligneous conjunctivitis, corneoscleral fibrous histiocytoma, and a corneal burn, were treated with AM and included in the veterinary literature.^8^, ^9^, ^10^, ^11^, ^12^, ^13^, ^14^, ^15^, ^16^

A multicenter retrospective study of cryopreserved AM transplantation used for the treatment of complicated corneal ulcers in 111 dogs (114 eyes) is the largest canine study to date and included eyes with keratomalacia (*n* = 51 eyes), stromal ulcers (*n* = 33), descemetoceles (*n* = 17), and corneal perforations (*n* = 13).^8^ Brachycephalic breeds accounted for 74/111 (66.6%) of the dogs. Cryopreserved human (*n* = 32 eyes) or bovine (*n* = 82) AM was applied using a monolayer (*n* = 31), bilayer (*n* = 44), or multilayer (*n* = 39) technique. The AM grafts were performed as a sole surgical technique and were anchored to the limbus (*n* = 52 eyes), the corneal defect (*n* = 48), or both (*n* = 14). In all the reported cases, the AM graft was placed epithelial side up (inlay technique). Microbiologic assessment of corneal samples was not reported. Mean epithelial healing time for all cases was 25.6 days (range 15–45 days), and mean case follow‐up times was 98.7 days (range 21–400 days).^8^ Graft failure occurred in five (4.4%) cases. Other reported complications included graft pigmentation (*n* = 10 eyes), suture dehiscence (*n* = 2), phthisis bulbi (*n* = 2), keratoconus (*n* = 1), epithelial inclusion cyst (*n* = 1), and anterior synechia (*n* = 1). Postsurgical complications were statistically associated with descemetoceles/perforations, use of human AM graft material, concurrent ocular diseases, and larger corneal defects. Good cosmetic and visual outcomes were achieved in 113 (99.1%) and 111 (97.4%) of cases, respectively. The size of corneal defect was statistically associated with poor or absent vision, with a median defect diameter of 5 mm in visual eyes and 9 mm in eyes with poor or absent vision.^8^

A case series of 40 dogs (41 eyes) and 5 cats (5 eyes) described the use of a low‐temperature vacuum‐dehydrated human AM product (i.e., Omnigen®, NuVision Biotherapies Ltd., Nottingham, UK).^9^ The AM graft was utilized as a sole procedure in five eyes (epithelial side facing up), as a supplementary graft combined with another biomaterial graft in 29 eyes (epithelial side facing up), and as a “patch” (epithelial side facing down) sole procedure or overlying another graft in 12 eyes. When the AM was used as a supplementary graft or patch, the additional graft materials included porcine urinary bladder submucosa in 33 eyes and frozen donor cornea in 7 eyes.^9^ Reported case details were not presented by animal species, but treated ocular lesions included 25 corneal stromal ulcers (i.e., mid‐ and deep stromal ulcerations), 14 corneal perforations, 5 descemetoceles, 1 corneal laceration, and 1 corneal sequestrum. Brachycephalic breeds accounted for 37/45 (82.2%) of the cases. Bacterial cultures were performed in 19 eyes, and culture results were described for 8 eyes. Bacteria isolated included *Pseudomonas aeruginosa* (*n* = 2 isolates), *Pasteurella* spp. (*n* = 1), *Streptococcus canis* (*n* = 1), *Staphylococcus pseudintermedius* (*n* = 1), and *Escherichia coli* (*n* = 1). There was no growth from 4/8 (50%) of the samples. Fungi were identified by corneal histopathology in a single case. Case outcomes were not divided by species, but median epithelial healing time was reported as 19 days (range 8–67 days) and median clinical follow‐up time was 84 days (range 2–1026 days).^9^ Successful anatomical healing and globe retention occurred in 43/46 (93.5%) eyes, but some required additional surgical intervention. Graft failure occurred in 10/46 (21.7%) eyes and were attributed to complete graft dehiscence (*n* = 3 eyes), progressive keratomalacia (*n* = 3), corneal perforation under the graft (*n* = 3), and persistent aqueous humor leakage (*n* = 1). Graft failure requiring enucleation occurred in three eyes and was the result of persistent keratomalacia in two eyes where the AM was used as a supplementary graft and corneal perforation in one eye where the AM graft was performed as a sole procedure.^9^ Minor graft complications occurred in 10 of 46 operated eyes and included partial graft retraction (*n* = 4 eyes), corneal degeneration (*n* = 2), anterior synechiae (*n* = 2), secondary glaucoma due to extensive anterior synechiae (*n* = 1), and development of an inclusion cyst within the graft site (*n* = 1). Thirty‐one of 33 (93.9%) operated eyes were visual at the final re‐examination, but this number excluded 3 enucleated eyes and 10 eyes with unknown visual status in the medical record. Blindness was attributed to extensive corneal vascularization and retinal degeneration in the two nonvisual cases. Successful anatomical healing occurred in 43/46 (93.5%) eyes.^9^

The clinical outcomes of 21 dogs with complicated corneal defects repaired with cryopreserved canine AM and the impact of age of the donor from which the placenta was harvested and the duration of cryopreservation was evaluated.^10^ Storage period was defined as short‐term (2–50 days), middle‐term (92–210 days), and long‐term (256–357 days). Corneal perforations (*n* = 11 eyes), descemetoceles (*n* = 8), and deep stromal corneal ulcers (*n* = 2) were included. Brachycephalic breeds accounted for 15/21 (71.4%) of the dogs. Microbiologic assessment of corneal samples was not reported. One to three layers of AM were applied. Amniotic membrane was placed with the epithelial side facing up when a single layer of AM was used. In the cases where two AM layers were used, the first layer was placed with the epithelium face down and the second layer was applied with the epithelial side facing up. In corneas treated with three layers of AM, the first and the second layers were secured with the epithelial side facing down and the third layer was applied with the epithelium face up. Mean corneal epithelization time in all dogs was 12 (+/− 3.9) days.^10^ Corneal defects repaired with AM stored for a shorter period healed sooner than the defects repaired with AM stored for middle and longer periods. Vision was present in 18/21 cases (85.7%), and the presence of vision did not correlate with the storage time of the AM used. The grafted area of the cornea was subjectively considered mildly opaque (*n* = 2 eyes), moderately opaque (*n* = 7), and severely opaque (*n* = 11) at postoperative Day 60. Corneal opacification scores did not differ significantly between the storage period groups. Biochemical evaluation of the stored amnion determined that tissue inhibitor of matrix metalloproteinase‐1 concentrations significantly decreased over a year of storage time, but total protein and hyaluronic acid concentrations did not change.^10^

A case series described the use of cryopreserved equine AM grafts in eight dogs with variety of types of corneal ulcerations, including indolent corneal ulcers and corneal ulcers associated with keratoconjunctivitis sicca.^11^ Microbiologic assessment of corneal samples was not reported. Several different surgical approaches were employed in the cases. Grafts were maintained for at least 12 days in nine eyes (in one case the graft was missing after 5 days).^11^ The clinical results associated with the grafts were subjectively described as good in most cases with corneal healing by postoperative Day 15. In one case where the entire corneal surface was covered by a single layer of AM after keratectomy, the authors noted no subjective benefit of the AM and corneal granulation tissue developed resulting in a worsened clinical situation.^11^

Additional smaller case series and case reports describe the use of AM grafts in the treatment of 12 cases of canine ocular surface disease. Frozen canine AM grafts (oriented with the epithelial side facing up) were placed over the entire cornea concurrent with a third eyelid flap after limbal dermoid removal by keratoconjunctivectomy in seven dogs.^12^ Epithelization of the keratectomy sites was complete between 7 and 14 days in all operated dogs and what was described as normal corneal transparency was reestablished between 3 and 8 weeks in all described cases.^12^ Cryopreserved or glycerin‐preserved canine AM grafts (graft orientation was not described) were used in the successful surgical treatment of severe keratomalacia in a dog and during the surgical excision of a canine corneoscleral fibrous histiocytoma.^13^ Cryopreserved equine AM and an undescribed type of AM were used during the repair of superficial lamellar keratectomy wounds (AM graft orientation was not described) performed to remove corneal epithelial inclusion cysts in two dogs.^14^, ^15^ Transplantation of cryopreserved equine AM (overlay technique) was used to repair the ocular surface after pseudomembrane excision in a Doberman Pinscher with ligneous conjunctivitis.^16^

Generally, the clinical success of AM grafting in the summarized veterinary literature was reported to be anatomically acceptable, achieving a good cosmesis, or was implied in less well‐defined terms, in 93%–100% of the eyes, while vision, when applicable, was reported to be maintained or restored in 85%–93% of cases.^8^, ^9^, ^10^, ^11^, ^12^, ^13^, ^14^, ^15^, ^16^ The indications, types of AM, surgical techniques used, postoperative follow‐up periods, and reporting styles varied widely between publications, precluding further analyses of clinical outcomes.

### 2.2 Porcine urinary bladder acellular matrix (ACell®)

Porcine urinary bladder acellular matrix (UBM) is a lyophilized and dehydrated extracellular matrix product derived from the lamina propria and basement membrane of the porcine urinary bladder. It is comprised of collagen and growth factors. It serves as a scaffolding that promotes cellular proliferation, integration, and tissue regeneration and is ultimately degraded and replaced by host tissue.^17^ It is supplied as 15 mm corneal discs and sheets of various sizes (ACell® Vet, Integra LifeSciences Corp, Princeton, NJ). There is not an up or down side orientation for these materials.

A total of 87 eyes from dogs with a variety of ophthalmic diseases, including corneal ulcers and perforations, that were treated with porcine UBM are included in the veterinary literature. In addition, there are reports of 36 conjunctival pedicle grafts and 10 CLCTs that were used in combination with porcine UBM.

A retrospective study of 38 eyes from 37 dogs evaluated the use of porcine UBM alone for corneal reconstruction.^18^ A lamellar keratectomy was performed to remove the diseased area of cornea, and a single layer of ACell® was sutured into the defect. Treated corneal lesions included corneal perforations (*n* = 21 eyes), deep corneal ulcerations without keratomalacia (*n* = 6), deep corneal ulcerations with keratomalacia (*n* = 4), and descemetoceles (*n* = 7). Brachycephalic breeds accounted for 32/37 (86.5%) of the dogs. Microbiologic assessment of corneal samples was not reported. Graft dehiscence or sloughing occurred in eight dogs.^18^ Vision was maintained in 22/30 (73%) eyes, but these numbers excluded eyes that developed glaucoma, phthisis bulbi, were lost to follow‐up or were enucleated. All dogs developed moderate‐to‐severe corneal scarring after surgery, and eight eyes had scarring that prevented visualization of the anterior chamber through the grafted area.^18^

A retrospective study evaluated the comparative success rates of conjunctival pedicle flaps with or without a concurrent underlying UBM graft in dogs.^19^ The study population consisted of 73 eyes from 69 dogs, including 37 eyes receiving conjunctival pedicle flaps alone and 36 eyes receiving a conjunctival pedicle flap with an ACell® or porcine small intestinal submucosa (described later) graft. Operated corneal lesions included corneal perforations (*n* = 36 eyes), descemetoceles (*n* = 19), deep stromal ulcers (*n* = 17), and unrecorded (*n* = 1).^19^ Brachycephalic breeds accounted for 55/69 (79.7%) of the dogs. Microbiologic assessment of corneal samples was not reported. The overall success rate for eyes that received a conjunctival pedicle flap was 36/37 (97%) and 32/36 (89%) for eyes that received a conjunctival pedicle flap with an ACell® or porcine small intestinal submucosa graft. These outcome results were not significantly different.^19^

The use of porcine UBM for the surgical reconstruction of the cornea in 28 eyes from 27 dogs with corneal ulcers was described.^20^ Brachycephalic breeds accounted for 23/27 (85.2%) of the dogs. Circular lamellar keratectomy was preformed to remove collagenolytic tissue, and one or two layers of ACell® graft were sutured into the defect.^20^ Corneal bacterial cultures were positive in nine dogs (e.g., *Streptococcus* spp., *Escherichia coli*, and *Pseudomonas aeruginosa* cultured from the cases) and negative in 16 dogs. The overall success rate for globe and vision retention was 100% at 90 days after surgery. One case required an additional corneal graft after the first surgery due to progression of keratomalacia. The degree of corneal opacity was scored as severe in 3/28 eyes (11%), moderate in 21/28 eyes (75%), and mild in 4/28 eyes (14%).^20^

A retrospective study described the use of porcine UBM concurrent with corneoconjunctival transposition (CCT) surgery (*n* = 10 eyes) for the treatment of deep corneal ulcers and compared outcomes to cases treated with CTT alone (*n* = 9).^21^ A single layer of ACell® was sutured into the ulcer bed and covered by the CCT for the combined treatment cases. Corneal lesions treated included corneal perforations (*n* = 12 eyes) and deep corneal ulcers including descemetoceles (*n* = 7). Brachycephalic breeds accounted for 13/18 (72.2%) of the dogs. All cases had bacterial and fungal cultures performed. Eight bacterial cultures were positive. Bacterial isolates included *Staphylococcus* spp. (*n* = 5), *Streptococcus* spp. (*n* = 3), *Pseudomonas aeruginosa* (*n* = 2), and *Klebsiella pneumoniae* (*n* = 1), but the isolates belonging to cases that received ACell® concurrent with CTT were not specified.^21^ Short‐term postoperative complications included cornea granulation tissue formation (*n* = 19 eyes), severe corneal edema (*n* = 4), graft retraction (*n* = 4), and anterior synechia (*n* = 1). Vision was present in 17 of 19 eyes (89.5%) at the final evaluation and 2 eyes were blind due to cataract formation. There were no statistical differences detected in the frequency of short‐term or long‐term postoperative complications between eyes that received ACell® and eyes that did not.^21^

Generally, success for reconstructions using porcine UBM alone were reported as globe retention, healed or in less well‐defined terms, ranging from 89% to 100% of the eyes, while vision, when applicable, was reported to be present/retained in 69%–98% of cases.^18^, ^19^, ^20^, ^21^ Despite the high success rate, some studies reported a relatively low percentage of cases having only mild opacification. The indications, surgical techniques used, postoperative follow‐up periods, and reporting styles varied widely between publications, precluding further analyses of clinical outcomes. It is noteworthy that the reported success rates of the reconstructions with either CCT or pedicle grafts that were combined with porcine UBM were not statistically different to the reconstructions made without porcine UBM.^21^

### 2.3 Porcine small intestinal submucosa

Porcine small intestinal submucosa (SIS) is a biodegradable, collagen‐based material derived from the submucosal layer of porcine jejunum. It is composed of three layers: tunica muscularis mucosa, tunica submucosa, and the stratum compactum layer of the tunica mucosa. Porcine SIS is acellular, nonimmunogenic, and acts as a three‐dimensional scaffold for tissue repair and remodeling. It is available as both sheets and discs of various sizes (Vetrix® BioSIS, Cumming, GA). The mucosal side of the graft is smooth and the serosal side is rough, but there is currently no evidence that orientation plays a role in the migration of cells across or into the material.

A total of 88 eyes from dogs with a variety of ophthalmic diseases, including multiple types of corneal ulcers and perforations, and surgical reconstructions of 3 eyes with limbal melanocytomas and 2 eyes with epithelial inclusion cysts, that were treated with porcine SIS are included in the veterinary literature.

The largest retrospective study published to date evaluating porcine SIS for corneal repair described the efficacy of SIS in corneal reconstructive surgery in 60 dogs (60 eyes).^22^ The dogs had keratomalacia (*n* = 42 eyes), severe corneal injury (*n* = 17), and limbal melanocytoma (*n* = 1), including 16 eyes with corneal perforations and 7 eyes with descemetoceles. Brachycephalic breeds accounted for 40/60 (66.7%) of the dogs. One to six layers of porcine SIS were used in each case with the mean number of SIS layers being 3.62 for all cases. Microbiologic assessment of corneal samples was not reported. Cases were followed for ≥3 months after surgery.^22^ Postoperative complications 3 months after surgery included corneal pigmentation in 19 dogs (31.6%). All cases were visual at 3 months after surgery. Corneal scarring at 3 months after surgery was described as transparent or mild in 54/60 (90%) eyes and marked in six eyes. A subset of cases was followed for a period greater than 3 months and severe, vision‐impairing corneal pigmentation developed in five eyes.^22^

The efficacy of a four‐layer porcine SIS graft (Vetrix BioSIS plus+) graft for the treatment of deep corneal lesions in 10 dogs with corneal perforations (*n* = 4 eyes), descemetoceles (*n* = 3), limbal melanocytomas (*n* = 2), and a deep corneal ulcer (*n* = 1) is described.^23^ Brachycephalic breeds accounted for 6/10 (60%) of the dogs. All corneal ulcers had bacterial and fungal cultures performed. Bacterial cultures were positive in each case and *Pseudomonas aeruginosa, Staphylococcus* spp., *Streptococcous* spp., and *Enterobacter* spp. were isolated. Mean postsurgical follow‐up length was 86 days.^23^ Mild postoperative complications include partial keratomalacia (*n* = 3 cases), mild corneal pigmentation (*n* = 1), and anterior synechia (*n* = 1). Twelve of 13 eyes (92.3%) were visual at the final evaluation, and one case developed severe keratomalacia requiring enucleation 21 days after surgery.^23^

A case series described the use of porcine SIS grafts in five brachycephalic dogs with deep melting corneal ulcers.^24^ Corneal bacterial cultures were performed for all dogs and were positive for bacterial growth in a single case (*Pseudomonas aeruginosa* and *Staphylococcus pseudintermedius* isolated). All cases had ≥6 months of follow‐up time.^24^ At 15 days post‐surgery, all corneas were re‐epithelialized and superficial intense corneal vascularization surrounded the graft. After 4 weeks, the SIS graft was opaque in all cases with associated corneal vascularization that progressively decreased after 45 days. No significant postoperative complication were described. At 6 months after surgery, four of five eyes (80%) had complete corneal transparency restored and vision was preserved in all five cases.^24^

A retrospective study was performed to evaluate the efficacy of porcine SIS grafting covered by a conjunctival flap for the surgical repair of full‐thickness corneal wounds in several species including six dogs.^25^ Dog operated included corneal perforations (*n* = 5 dogs) and a limbal melanocytoma (*n* = 1). Brachycephalic breeds accounted for 4/6 (66.7%) of the dogs. All corneal perforations had bacterial cultures performed, and these were positive for growth in three eyes. Cultured bacteria included *Pseudomonas aeruginosa* (listed as “*Pasteurella aeruginosa*” in the manuscript), *Staphylococcus* spp., *Streptococcus* spp., and *Escherichia coli*. Cases had at least 2–5 months follow‐up time.^25^ Partial graft dehiscence and aqueous humor leakage requiring a second surgery were reported as short‐term complications in one dog. All cases were visual at the final follow‐up examination, except a single dog that developed severe corneal pigmentation.^25^

Several additional smaller case series and case reports describe the successful use of porcine SIS grafts in the treatment of canine ocular surface disease. These cases include corneal reconstruction following keratectomy for corneal epithelial inclusion cysts and in conjunction with a conjunctival graft for repair of a full‐thickness corneoscleral defect resulting from limbal melanoma excision in a dog.^15^, ^26^ Four dogs with deep corneal ulcers were successfully treated with suture‐less porcine SIS grafts performed in combination with a nictitating membrane flap.^27^

In general terms, surgical success was reported in 80%–100% of the eyes, while vision, when applicable, was maintained or restored in 80%–100% of cases, though some studies that reported good transparency in as few as 70% of cases reported vision in 100% of cases, demonstrating the intrinsic difficulties and crudeness of vision assessment in veterinary ophthalmology.^22^, ^23^, ^24^, ^25^, ^26^, ^27^ The variety of indications, surgical techniques used, postoperative follow‐up periods, and reporting styles varied widely between publications, precluding further analyses of clinical outcomes.

### 2.4 Acellular porcine corneal stroma (BioCorneaVet™)

Acellular porcine corneal stroma (APCS) is a relatively new biomaterial available under the tradename BioCorneaVet™ (Xeno Surgical, Beijing, China). BioCorneaVet™ is a processed and lyophilized porcine stroma that is produced in 10 mm and 12 mm diameter discs, available in multiple graft thicknesses (150–600 μm), and it possess a relatively long shelf life. The APCS graft material is thicker and stiffer than many other biomaterials used for corneal reconstruction and possess improved handling characteristics for some surgical applications. Similar bioartificial porcine corneas when evaluated in dogs without ocular disease after experimental lamellar keratectomy displayed rapid corneal re‐epithelialization and the restoration of corneal transparency and normal histologic characteristics over several months.^28^, ^29^

A total of 45 canine eyes with deep or perforating corneal ulcers that were treated with APCS grafts are included in the veterinary literature.

A prospective pilot study evaluated the use of APCS in dogs undergoing penetrating keratoplasty for the repair of deep corneal ulcers included three descemetoceles and two corneal perforations.^30^ Brachycephalic breeds accounted for 4/5 (80%) of the dogs. Microbiologic assessment of corneal samples was not reported. All cases had a minimum of 10 months postoperative follow‐up time. Corneal re‐epithelialization was complete in the majority of grafts after 1 month. Focal graft dehiscence and retraction occurred in a single case 2 weeks after surgery, but it did not require additional surgical intervention.^30^ All grafts survived until final examination with a stable anterior chamber. Most APCS grafts became diffusely vascularized between 2 and 4 weeks after surgery; then, the vessels started to regress. By 4–6 months after surgery, two dogs had minimal‐to‐mild graft opacification, two dogs had moderate graft opacification, and one dog had severe graft opacification. During re‐examinations at 4–6 months, corneal opacification continued to progressively improve in all but one of the dogs.^30^

A retrospective study described the outcome of corneal grafting with BioCorneaVet™ in dogs with deep corneal ulcerations.^31^ Forty dogs were evaluated, including 25 corneal perforations, 8 descemetoceles, and 8 deep stromal defects. Brachycephalic breeds accounted for 34/40 (85%) of the dogs. Bacterial culture was performed 13/40 cases (32.5%) with negative results in 6/13 cases (46.15%) and positive in results in 7/13 cases (53.84%). The isolated bacteria were *Pseudomonas* spp. (*n* = 2 cases), *Escherichia coli* (*n* = 2), *Chryseobacterium* spp. (*n* = 1), *Streptococcus canis* (*n* = 1), and a single mixed infection of an *Enterococcus* spp. and *Escherichia coli*. Cases had a mean follow‐up time of 233 days (range: 28–797 days).^31^ Corneal APCS graft re‐epithelialization was complete in a median of 10 days (range 4–19 days). Postoperative complications included mild‐to‐severe corneal vascularization in all cases, partial graft dehiscence in three cases (7.5%), graft infection and keratomalacia in two cases (5%), and glaucoma in two cases (5%). Ocular integrity was maintained in 37 cases (92.5%), and vision was preserved in 36 cases (90%). Three eyes (7.5%) ultimately required enucleation for glaucoma or subsequent corneal perforation, and a single eye was nonvisual from development of severe corneal pigmentation.^31^

The main reported clinical indications for BioCorneaVet™ reported to date include deep or perforated corneal ulcers in dogs.^30^, ^31^ Generally, surgical success was reported in 92%–100% of the eyes, while vision, when applicable, was maintained or restored in 90%–100% of cases.

### 2.5 Miscellaneous soft tissue and cartilage grafts

Several additional biological grafts are reported for corneal reconstruction in dogs, including preserved equine renal capsule, autologous omentum, autologous buccal mucosa membrane, bovine pericardium, and homologous peritoneum.

An experimental study was performed to evaluate the use of equine renal capsule preserved in 98% glycerine to repair lamellar corneal lesions in normal dogs.^32^ A 5.0 mm diameter corneal trephine was used to create a half‐thickness defect in the cornea, and the renal capsule was sutured into the defect. Clinical and histological evaluations were performed between 1 and 60 days after surgery. Corneal re‐epithelialization was complete by 4 days after surgery in all dogs. Marked corneal edema developed immediately after surgery and then gradually reduced.^32^ Corneal vascularization was noted on postoperative Day 7 and persisted until study conclusion. Histopathologic findings included inflammatory cell accumulation at the junction of the graft and host tissue. Dense corneal fibrosis developed in the region of the grafts but became semitransparent by the study's conclusion.^32^

An experimental study evaluated the effects of omental grafting in dogs with corneal alkali injuries.^33^ Omental elongation and pedicle transposition through a subcutaneous tunnel was performed, with preservation of the vascular supply, immediately following induction of an ocular surface alkaline burn. Dogs with omental grafting were compared to dogs that received medical treatment only, and they were followed for 6 months after surgery.^33^ At 6 months after surgery, corneal opacification and vascularization were significantly reduced in dogs that received omental grafts. Histopathologic evaluation of the corneas revealed restoration of the corneal epithelium and stroma in dogs with the omental grafts with relatively few vessels noted, while the control groups exhibited corneal conjunctivalization and marked stromal vascularization.^33^

A total of 20 canine eyes that were clinically treated with an either autologous buccal mucous membrane, bovine pericardium, or homologous peritoneum grafts are included in the veterinary literature.

A retrospective study evaluated the efficacy, outcomes, and complications of autologous buccal mucous membrane grafts for the repairs of severe corneal ulcers in 14 dogs.^34^ The study included corneal perforations (*n* = 11 eyes), descemetoceles (*n* = 2), and a deep stromal corneal ulcer (*n* = 1). Brachycephalic breeds accounted for 12/14 (85.7%) of the dogs. Buccal mucous membrane grafts were harvested from the unpigmented superior labial mucosa using a biopsy punch and secured to the cornea. The grafts were then covered with a conjunctival pedicle graft in most eyes, and all eyes received a third eyelid flap. Microbiologic assessment of corneal samples was not reported. The median follow‐up period was 549.2 days (range 14–2691 days), but these numbers also included results for a group of cats that were described in the study.^34^ Globe retention was achieved in 12 of 14 eyes (85.7%) with vision present in all 11 eyes (78.6%). Two canine cases were enucleated (one for endophthalmitis and one for glaucoma), and one dog developed phthisis bulbi subsequent to endophthalmitis.^34^

The use of bovine pericardium grafts is described for the treatment of deep corneal ulcers with keratomalacia in three dogs.^35^ Keratectomy was performed to remove malacic corneal tissue, a corneal trephine was then used to prepare the pericardium graft (Tutopatch®; Tutogen Medical Inc., Metz, France), and the graft was sutured over the keratectomy bed. Bacterial corneal culture was performed for all cases, and *Pseudomonas aeruginosa* was isolated from two dogs and results were negative for the other dog. Dogs were followed for up to 6 months after surgery.^35^ Prominent corneal vascularization was present around the graft in all eyes at 1 week after surgery. All eyes were re‐epithelialized by 2 weeks after surgery. Two months after the surgery, two of three corneas had healed with focal corneal scarring. The remaining dog had progression of the keratomalacia that required additional surgery but became blind due to corneal opacification.^35^

A small case series described the use of bovine pericardium to repair ocular surface lesions in two dogs, including a penetrating surgical defect created by limbal melanoma excision and a traumatic full‐thickness corneal laceration.^36^ Both cases were considered successful by the authors with maintenance of vision. Residual corneal opacification at the graft site was present during the final examination (i.e., 4 to 18 months, respectively) in both cases.^36^

A preserved homologous peritoneum graft was used to repair a congenital scleral staphyloma in a dog.^37^ A sheet of homologous peritoneum preserved in glycerol was sutured over the bulging scleral tissue and covered with a conjunctival flap. At 3 months after surgery, the graft was well integrated into the ocular surface and the size of the staphyloma was reduced.^37^

Generally, success was reported as maintenance of the globe in 83%–89% of the eyes, while vision, when applicable, was reported as maintained or restored in approximately 81%–83% of cases.^34^, ^35^, ^36^, ^37^ This group of biomaterials contained the lowest number of eyes treated and, therefore, the results must be interpreted with caution.

### 2.6 Keratoprosthesis

Keratoprosthesis (KP) surgery, or artificial corneal transplant, is indicated in human patients for severe, vision‐compromising corneal disease that is not amenable to other keratoplasty techniques.^38^ Although KP provides an opportunity to restore vision in cases where no other options exist, these procedures are considered technically demanding, require intensive and long‐term postoperative care, and are associated with relatively high complication rates (e.g., infection, glaucoma, and implant extrusion).^38^ Numerous types of KP implants are describe for use in human patients.^39^

Publications describing KP surgery in veterinary ophthalmology are currently limited.^40^, ^41^, ^42^ A total of 28 canine eyes with near or complete corneal blindness treated by KP surgery are included in three separate publications in the veterinary literature.

A case series described the use of a novel, one‐piece, silicone KP prototype in seven nonbrachycephalic dogs with corneal blindness.^40^ Operated dogs were affected by severe endothelial disease (*n* = 5 dogs) or chronic superficial keratitis (*n* = 2). Surgical implantation of the prosthesis was performed just anterior to Descemet's membrane in a stromal pocket. An intraoperative corneal perforation occurred during implantation in one dog, but the other surgeries were regarded as uneventful. All described eyes regained limited vision immediately after surgery. Serious postoperative complications developed in 5/7 (71.4%) eyes including one KP implant extrusion after 8 weeks and purulent keratitis in 4/6 (66.7%) of the remaining eyes between 3 and 6 months postoperatively, necessitating implant removal with associated vision loss.^40^ Bacterial and fungal cultures were negative in all cases, but at the time of implant extraction it was noted that the implant was not integrated into the adjacent corneal stroma and was accumulating debris.^40^ The remaining two cases with intact KP implant had presented with chronic superficial keratitis and maintained vision for ≥12 months.^40^

A retrospective clinical study that evaluated a penetrating KP procedure using a posterior fixation implant made of polymethylmethacrylate and polytetrafluoroethylene that was designed to be maintained against the posterior corneal surface by the intraocular pressure (PCL5® Corneal S.A., Paris, France) is the largest canine KP series published.^41^ Twenty eyes, from 19 dogs, with total corneal opacification resulting from chronic superficial keratitis (*n* = 11 eyes), keratoconjunctivitis sicca (*n* = 5), endothelial dystrophy (*n* = 3), and chemical keratitis (*n* = 1) were operated. Brachycephalic breeds accounted for 5/19 (26.3%) of the dogs. A successful outcome with maintenance of vision and no substantial postoperative complications occurred in 6/20 (30%) eyes. All successful KP cases had a minimum of 8 months postoperative follow‐up time. Enucleation was performed between 1 and 8 weeks after surgery in 5/20 eyes (25%) for prosthesis extrusion (three eyes also developed bacterial endophthalmitis).^41^ Other complications included development of a retroprosthetic membrane {5/20 eyes (25%)} in the first 30 days after surgery that limited vision and required surgical excision, and overgrowth of granulation tissue the progressively covered the KP optic {4/20 eyes (20%)} in the first 4–12 weeks following the surgery requiring surgical removal to restore vision.^41^

An additional case report described the use of a polymethylmethacrylate KP for blinding chronic immune‐mediated superficial keratitis in a single dog.^42^ The short‐term outcome of the surgery appeared good, but the report only included 30 days of postoperative follow‐up time.^42^

The overall reported success with KP implants in dogs is low with a total of 19/28 (48.3%) of the eyes included having implant failure.^40^, ^41^, ^42^ All the eyes were blinded due to extensive ocular surface disease prior to surgery and approximately half of the eyes had partial or complete restoration of vision for the follow‐up times included. Despite this, the number of patients is too low to draw significant conclusions and additional investigation of KP implant and surgical techniques for dogs is warranted.

## 3 CONCLUDING COMMENTS

Animal, disease, and surgical factors impacting graft survival and ultimate corneal clarity following corneal reconstructive surgery remain poorly understood in veterinary ophthalmology. As described in the present review, inconsistent study design and data reporting make direct comparison between studies, publications, and graft types challenging. Variables potentially affecting surgical outcomes extend beyond the choice of graft type and surgical technique and could include other factors such as animal signalment, concurrent ocular or systemic diseases, microbiologic details, suture material utilized, and postoperative medication selection.

Future studies that are designed and executed to specifically compare corneal reconstructive techniques are needed to improve surgical results. These studies could utilize advanced anterior segment ocular imaging techniques (e.g., in vivo confocal microscopy, optical coherence tomography, Scheimpflug corneal tomography, specular microscopy, and ultrasound biomicroscopy) and newer clinical microbiologic technologies (e.g., next‐generation sequencing) to improve the understanding of these elements and how they can be manipulated to enhance outcomes. Surgical outcome variables that have received little attention in previous studies, such as the restoration of normal corneal thickness, corneal refractive state, preservation of the corneal endothelium, and maintenance of corneal sensitivity, could be evaluated in future investigations and might lead to refinements in clinical practice.

## AUTHOR CONTRIBUTIONS

The development of the idea as well as the writing and editing was carried out by the authors. All of the authors contributed equally to this manuscript.

## CONFLICT OF INTEREST STATEMENT

The authors confirm there are no conflicts of interest.

## ETHICS STATEMENT

This study complies with the Guidelines for Ethical Research in Veterinary Ophthalmology (GERVO) and is exempt from approval by an ethics committee. The client owned patient images were taken with written permission of the client.

## Data Availability

Data sharing is not applicable to this article as no datasets were generated or analyzed during the current study. All the reviewed articles are part of the veterinary and medical literature.

## References

[1] Chen Z , You J , Liu X , et al. Biomaterials for corneal bioengineering. Biomed Mater. 2019;13(3):e032002. 10.1088/1748-605X/aa92d2 29021411

[2] Baum J . Thygeson lecture. Amniotic membrane transplantation: why is it effective? Cornea. 2002;21(4):339‐341. 11973378 10.1097/00003226-200205000-00001

[3] Malhotra C , Jain AK . Human amniotic membrane transplantation: different modalities of its use in ophthalmology. World J Transplant. 2014;4(2):111‐121. 25032100 10.5500/wjt.v4.i2.111 PMC4094946

[4] Godoy CA , Guerra JL , Barros PSM . Lamellar keratoplasty in dogs using equine fetal membrane as graft: experimental study. Arq Bras Oftalmol. 2002;65:545‐549.

[5] Tsuzuki YK , Izumisawa Y , Kotani T . Microstructure and glycosaminoglycan ratio of canine cornea after reconstructive transplantation with glycerin‐preserved porcine amniotic membranes. Vet Ophthalmol. 2009;11(4):222‐227. 10.1111/j.1463-5224.2008.00629.x 18638347

[6] Kim JY , Choi YM , Jeong SW , Williams DL . Effect of bovine freeze‐dried amniotic membrane (Amnisite‐BA) on uncomplicated canine corneal erosion. Vet Ophthalmol. 2009;12(1):36‐42. 19152596 10.1111/j.1463-5224.2009.00671.x

[7] Barros PSM , Garica JA , Laus JL , Ferreira AL , Salles Gomes TL . The use of xenologous amniotic membrane to repair canine corneal perforation created by penetrating keratectomy. Vet Ophthalmol. 1998;1(2–3):119‐123. 11397220 10.1046/j.1463-5224.1998.00026.x

[8] Costa D , Leiva M , Sanz F , et al. A multicenter retrospective study on cryopreserved amniotic membrane transplantation for the treatment of complicated corneal ulcers in the dog. Vet Ophthalmol. 2019;22(5):695‐702. 30716187 10.1111/vop.12643

[9] Maini S , Hurley‐Bennett K , Dawson C . Case series describing the use of low‐temperature vacuum‐dehydrated amnion (Omnigen) for the treatment of corneal ulcers in cats and dogs: 46 cases (2016–2017). Top Companion Anim Med. 2020;41:100474. 32919060 10.1016/j.tcam.2020.100474

[10] Dower NMB , Ribeiro AP , Gomes LG , et al. Concentrations of tissue inhibitor of matrix metalloproteinase‐1 and hyaluronic acid in canine amniotic membranes cryopreserved for different time points and its effects in dogs with complicated corneal ulcers. Vet Ophthalmol. 2022;25(1):62‐72. 34240563 10.1111/vop.12916

[11] Aracelli A , Tibaldini P , Angeli G , Bellezza E . Equine amniotic membrane transplantation in some ocular surface diseases in the dog and cat: a preliminary study. Vet Res Commun. 2009;33:S169‐S171. 10.1007/s11259-009-9284-6 19655266

[12] Kalpravidh M , Tuntivanich P , Vongsakul S , Sirivaidyapong S . Canine amniotic membrane transplantation for corneal reconstruction after the excision of dermoids in dogs. Vet Res Commun. 2009;33(8):1003‐1012. 19760128 10.1007/s11259-009-9319-z

[13] Barros PSM , Safatle AM , Godoy CA , Souza MS , Barros LF , Brooks DE . Amniotic membrane transplantation for the reconstruction of the ocular surface in three cases. Vet Ophthalmol. 2005;8(3):189‐192. 15910372 10.1111/j.1463-5224.2005.00391.x

[14] Choi US , Labelle P , Kim S , et al. Successful treatment of an unusually large corneal epithelial inclusion cyst using equine amniotic membrane in a dog. Vet Ophthalmol. 2010;13(2):122‐125. 20447032 10.1111/j.1463-5224.2010.00765.x

[15] Cassagnes C , Cognard SA , Nicolier A , et al. Corneal epithelial inclusion cysts in 12 dogs (13 eyes) from 2010 to 2019: a multicentric retrospective study. Vet Ophthalmol. 2020;23(5):856‐862. 32738182 10.1111/vop.12809

[16] O'Leary L , Specht A , Isaza N , et al. Amniotic membrane transplantation for ligneous conjunctivitis in a Doberman. Vet Ophthalmol. 2019;21(6):652‐660. 10.1111/vop.12559 29482261

[17] Mancuso LA , Lassaline M , Scherrer N . Porcine urinary bladder extracellular matrix grafts (ACell vet® corneal discs) for keratomalacia in 17 equids (2012–2013). Vet Ophthalmol. 2014;19(1):3‐10. 25429917 10.1111/vop.12240

[18] Chow DWY , Westermeyer HD . Retrospective evaluation of corneal reconstruction using ACell vet™ alone in dogs and cats: 82 cases. Vet Ophthalmol. 2016;19(5):357‐366. 26096693 10.1111/vop.12294

[19] Dorbandt DM , Moore PA , Myrna KE . Outcome of conjunctival flap repair for corneal defects with and without an acellular submucosa implant in 73 canine eyes. Vet Ophthalmol. 2015;18(2):116‐122. 25047064 10.1111/vop.12193

[20] Balland O , Poinsard AS , Famose F , et al. Use of porcine urinary bladder acellular matrix for corneal reconstruction in dogs and cats. Vet Ophthalmol. 2016;19(6):454‐463. 26559499 10.1111/vop.12326

[21] Keenan AV , Boveland SD , Rodriguez Galarza R , Moore PA . Corneoconjunctival transposition with and without ACell® for deep corneal ulcer repair in 18 dogs. Vet Ophthalmol. 2020;23(5):884‐891. 32790061 10.1111/vop.12815

[22] Goulle F . Use of porcine small intestinal submucosa for corneal reconstruction in dogs and cats: 106 cases. J Small Anim Pract. 2012;53(1):34‐43. 22122191 10.1111/j.1748-5827.2011.01149.x

[23] Barachetti L , Zanni M , Stefanello D , Rampazzo A . Use of four‐layer porcine small intestinal submucosa alone as a scaffold for the treatment of deep corneal defects in dogs and cats: preliminary results. Vet Rec. 2023;186(19):e28. 10.1136/vr.105513 31937546

[24] Vanore M , Chahory S , Payen G , Clerc B . Surgical repair of deep melting ulcers with porcine small intestinal submucosa (SIS) graft in dogs and cats. Vet Ophthalmol. 2007;10(2):93‐99. 17324164 10.1111/j.1463-5224.2007.00515.x

[25] Bussieres M , Krohne SG , Stiles J , Townsen WM . The use of porcine small intestinal submucosa for the repair of full‐ thickness corneal defects in dogs, cats and horses. Vet Ophthalmol. 2004;7(5):352‐359. 15310296 10.1111/j.1463-5224.2004.04055.x

[26] Lewin GA . Repair of a full thickness corneoscleral defect in a German shepherd dog using porcine small intestinal submucosa. J Small Anim Pract. 1999;40(7):340‐342. 10444755 10.1111/j.1748-5827.1999.tb03094.x

[27] Steinmetz A , Theyse LFH . Treatment of deep corneal ulcers with porcine small intestinal submusosa using a modified surgical technique in dogs. Clin Case Rep. 2020;9(2):812‐817. 33598250 10.1002/ccr3.3661 PMC7869326

[28] Hao Y , Zhou J , Tan J , et al. Preclinical evaluation of the safety and effectiveness of a new bioartificial cornea. Bioact Mater. 2023;29:265‐278. 37600931 10.1016/j.bioactmat.2023.07.005 PMC10432718

[29] Xu B , Song Z , Fan T . Construction of anterior hemi‐corneal equivalents using nontransfected human corneal cells and transplantation in dog models. Artif Organs. 2017;41(11):1004‐1016. 28621916 10.1111/aor.12878

[30] Lavaud A , Kowalska ME , Voelter K , Pot SA , Rampazzo A . Penetrating Keratoplasty in dogs using acellular porcine corneal stroma (BioCorneaVet™): a prospective pilot study of five cases. Vet Ophthalmol. 2021;24(5):543‐553. 33774897 10.1111/vop.12884

[31] Santillo D , Mathieson I , Corsi F , Göllner R , Guandalini A . The use of acellular porcine corneal stroma xenograft (BioCorneaVet™) for the treatment of deep stromal and full thickness corneal defects: a retrospective study of 40 cases (2019–2021). Vet Ophthalmol. 2021;24(5):469‐483. 34480395 10.1111/vop.12927

[32] Andrade A , Laus J , Figueiredo F . The use of preserved renal capsule to repair lamellar corneal lesions in normal dogs. Vet Ophthalmol. 1999;2(2):79‐82. 11397246 10.1046/j.1463-5224.1999.00052.x

[33] Shadmani A , Kazemi K , Khalili MR , Eghtedari M . Omental transposition in treatment of severe ocular surface alkali burn: an experimental study. Med Hypothesis Discov Inno Ophthalmol. 2014;3(2):57‐61. PMC4346679 25738161

[34] Mezzadri V , Crotti A , Nardi S , Barsotti G . Surgical treatment of canine and feline descemetoceles, deep and perforated corneal ulcers with autologous buccal mucous membrane grafts. Vet Ophthalmol. 2021;24(6):599‐609. 34085742 10.1111/vop.12907 PMC9292918

[35] Dulaurent T , Azoulay T , Goulle F , et al. Use of bovine pericardium (Tutopatch®) graft for surgical repair of deep melting corneal ulcers in dogs and corneal sequestra in cats. Vet Ophthalmol. 2014;17(2):91‐99. 23621151 10.1111/vop.12047

[36] Barros PSM , Safatle AMV , Malerba TA , Burnier JM . The surgical repair of the cornea of the dog using pericardium as a keratoprosthesis. Braz J Vet Res Anim Sci. 1995;32(4):251‐255.

[37] Barros PSM , Safatle AMV . Congenital scleral staphyloma in a dog repaired with preserved homologous peritoneum. Vet Ophthalmol. 2000;3(1):27‐29. 11397279 10.1046/j.1463-5224.2000.00102.x

[38] Koo EH , Hannusk SB . Challenges in management of the Boston Keratoprosthesis type 1. Curr Opin Ophthalmol. 2021;32(4):385‐388. 33973907 10.1097/ICU.0000000000000774

[39] Moshirfar M , Moody JJ , Barke MR , et al. The historical development and an overview of contemporary keratoprostheses. Surv Ophthalmol. 2022;67:1175‐1199. 35081413 10.1016/j.survophthal.2022.01.005

[40] Allgoewer I , McLellan GJ , Agarwal S . A keratoprosthesis prototype for the dog. Vet Ophthalmol. 2010;13(1):47‐52. 10.1111/j.1463-5224.2009.00759.x 20149176

[41] Isard PF , Dulaurent T , Regnier A . Keratoprosthesis with retrocorneal fixation: preliminary results in dogs with corneal blindness. Vet Ophthalmol. 2010;13(5):279‐288. 20840104 10.1111/j.1463-5224.2010.00800.x

[42] Isard PF , Mentek M , Dulaurent T . Keratoprosthesis in a dog with chronic immune‐mediated superficial keratitis. J Fr Ophtalmol. 2022;45(6):673‐674. 35537893 10.1016/j.jfo.2021.12.018

